# Context-sensitive attention is socialized via a verbal route in the parent-child interaction

**DOI:** 10.1371/journal.pone.0207113

**Published:** 2018-11-08

**Authors:** Moritz Köster, Joscha Kärtner

**Affiliations:** 1 Department of Psychology, University of Münster, Fliednerstraße 21, Münster, Germany; 2 Institute of Psychology, Free University Berlin, Habelschwerdter Allee 45, Berlin, Germany; University of Plymouth, UNITED KINGDOM

## Abstract

The way humans perceive and attend to visual scenes differs profoundly between individuals. This is most compellingly demonstrated for context-sensitivity, the relative attentional focus on focal objects and background elements of a scene, in cross-cultural comparisons. Differences in context-sensitivity have been reported in verbal accounts (e.g. picture descriptions) and in visual attention (e.g., eye-tracking paradigms). The present study investigates (1) if the way parents verbally guide the attention of their children in visual scenes is associated with differences in children’s context-sensitivity and (2) if verbal descriptions of scenes are related to early visual attention (i.e., gaze behavior) in 5-year-old children and their parents. Importantly, the way parents verbally described visual scenes to their children was related to children’s context-sensitivity, when describing these scenes themselves. This is, we found a correlation in the number of references made to the object versus the background as well as the number of relations made between different elements of a scene. Furthermore, verbal descriptions were closely related to visual attention in adults, but not in children. These findings support our hypotheses that context-sensitivity is socialized via a verbal route and that visual attention processes align with acquired narrative structures only later in development, after the preschool years.

## Introduction

Human basic cognitive functions, including scene perception and visual attention, differ profoundly between individuals, as revealed in cross-cultural research [1–3]. Specifically, Nisbett and Masuda [1] describe two prototypical attention styles; An analytic style with a focus on focal objects and their properties, being more typical for people from Western cultural contexts (low context-sensitivity), and a holistic style with a higher sensitivity for the context and the relations between elements in a scene, being more typical for East Asian adults (high context-sensitivity).

For example, using behavioral measures, Masuda and Nisbett [2] found that US-Americans tended to report and memorize a large focal fish swimming in an aquarium, while Japanese participants reported and remembered more details from the background, like plants and smaller animals. Furthermore, East Asians compared to Western People perceive more relations between different objects [4, 5] and are more easily deceived by optical illusions, for example, when adjusting a focal element within a deceptive context [6].

A similar pattern was reported in eye-tracking paradigms, investigating early visual attention processes [3, 7]. Chua and colleagues [3] presented pictures with a clear focal object and a background (e.g. a tiger in the woods) and recorded the gaze behavior of Chinese and US-American students. Chinese students spent more time looking on the background compared to US-American students. There is first evidence that cultural differences in verbal descriptions may be related to cultural differences in visual attention in adults [8], while no relations between eye-tracking and verbal and optical illusion measures were found for 5-year-olds from diverse cultural contexts [9].

Ontogenetically, context-sensitivity, as measured by behavioral tasks, undergoes a developmental change during the preschool years and the years thereafter [10, 11, 12]. Based on several classical behavioral measures (picture description, number of relations, optical illusions), Imada and colleagues [11] found consistent differences between Japanese and US children at 6 and 7 years. While this study found only slight differences in verbal accounts between 4 and 5 years of age (1 out of 3 measures), an earlier onset of cross-cultural differences in context-sensitivity was found for context dependent emotional judgements, already in the 4th year [12]. Furthermore, cultural differences between Japanese and US children have already been described for 3- and 4-year-olds in object recognition and object search measures [13, 14], and also in optical illusion tasks, when tested in traditional Himba children, where deception rates were very low compared to US children, already at 3 years of age [15]. It is assumed that the way parents’ verbally guide children’s attention plays a key role in the development of context-sensitivity [10, 16]. First empirical studies found cultural differences in the way mothers talk to their young infants [17] and when reminiscing visual scenes jointly with their 4- to 9-year-old children [18]. However, to test that context-sensitivity is socialized in the parent-child interaction, it is essential to assess verbal descriptions of parents and children independently, in order to avoid direct dyadic effects. Therefore, in the current study analyzed the way in which parents describe pictures for their children and children’s picture descriptions independently.

With regard to an early relation between verbal accounts of context-sensitivity and visual attention processes, a recent study by Köster and colleagues [19] found that verbal picture descriptions were associated with visual cortical processes (assessed with an electroencephalogram) in 7-year-olds, but not yet in 5-year-olds. Based on these findings, we hypothesized that context-sensitivity may be learned via a verbal route, before early visual processes reorganize and align with verbal measures of context-sensitivity, later in development.

**Fig 1:**
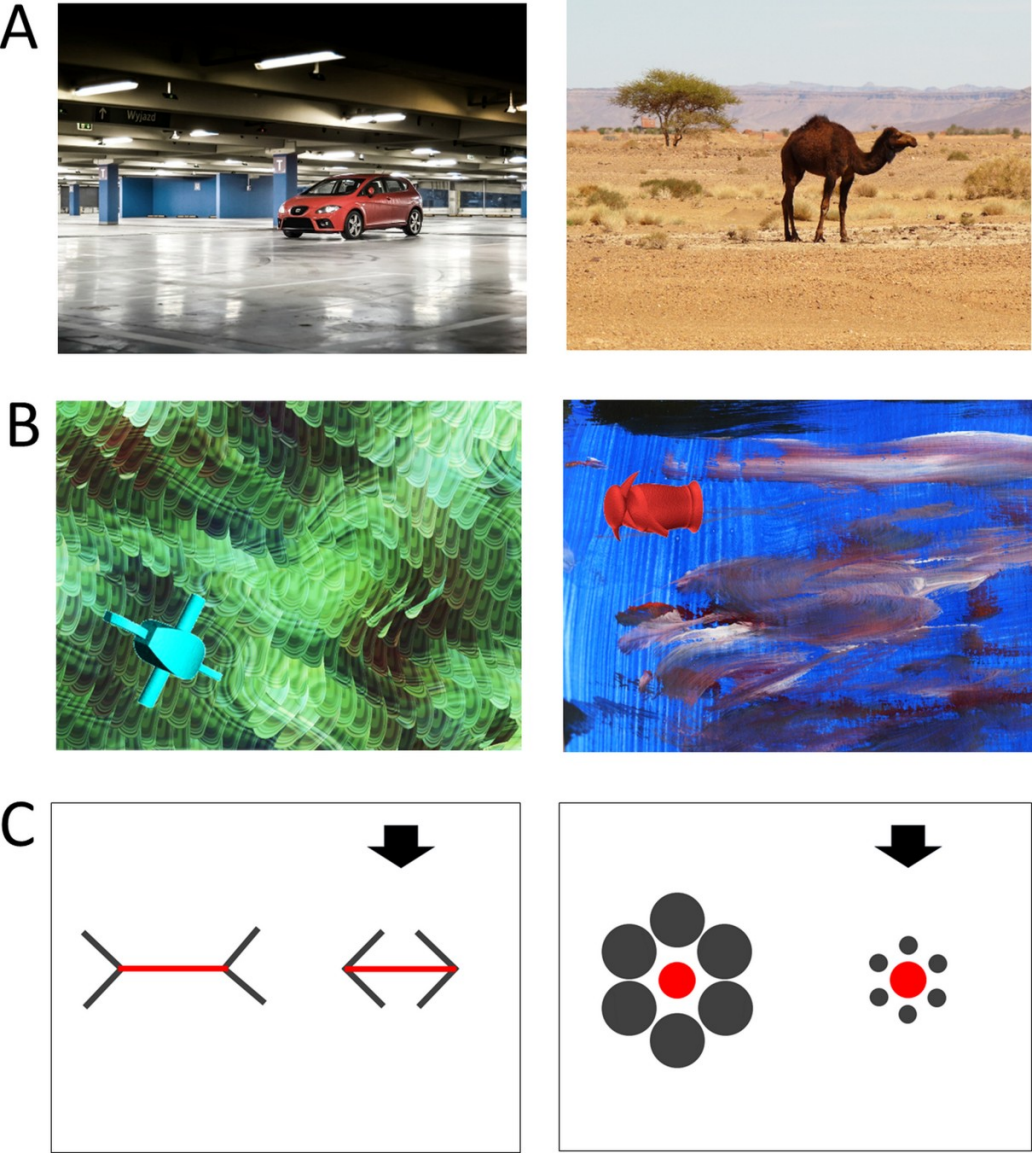
Example stimuli used in the experimental tasks. (A) Real pictures of scenes with a clear foreground and a clear background. (B) Abstract pictures, comprised of artificial objects (e.g., greebles and geons) and artworks or fractals as background. (C) Two of the four optical illusions used for the optical illusion task (left panel: Müller-Lyer illusion; right panel: Ebbinghaus illusion). The black arrows indicated, which red element could be adjusted. In the control condition the red elements were shown without gray context elements.

To test these hypotheses, we employed verbal measures of context-sensitivity (i.e., picture descriptions) and visual attention measures (i.e., gaze behavior) in children and their parents. Specifically, five-year-old children were asked to describe pictures with a focal object in front of a background and to watch a similar set of pictures, while their gaze behavior was recorded with an eye-tracker (Fig 1A). Thereafter, parents were asked to describe the same pictures to their children and to watch the same pictures like their children, while their gaze was recorded. Because visual attention may be influenced by the semantics of the visual scene (cf. [19]), we also included abstract, non-semantic stimuli in the eye-tracking assessments, to obtain a more objective measure of visual attention processes (Fig 1B). Note that we closely adapted the verbal measure from Imada and colleagues [10] and the eye-tracking paradigm from Chua and colleagues [3]. We also assessed children’s performance in an optical Illusion task, to test whether their verbal accounts of context-sensitivity would be related to a non-verbal behavioral measure (Fig 1C).

## Methods

### Participants

The sample consisted of 28 parents (24 mothers; *M*_age_ = 39;2 years; *SD*_age_ = 4;8 years, *Range*_age_ 28;0 – 46;0 years) and their preschool children (15 girls; *M*_age_ = 5;5 years; *SD*_age_ = 0;3 years, *Range*_age_ 5;1 – 6;1 years) from a German city. All participants had normal or corrected to normal visual acuity. Participants were recruited in collaboration with the city council. The study was carried out in accordance with the provisions of the World Medical Association Declaration of Helsinki. Informed written consent was obtained from the parent and children gave informed assent. Additional dyads did not complete all tasks and were thus excluded from further analysis. This was because children were not motivated to complete the data assessment (*n* = 4), procedural errors or technical problems occurring in the optical illusion task (*n* = 2), the picture description task (*n* = 1) or the eye-tracking task (*n* = 3), as well as slight squinting detected during the eye-tracking task in one child (*n* = 1).

### Ethics statement

This research was conducted in accordance with the Declaration of Helsinki and the Ethical Principles of the German Psychological Society (DGPs), the Association of German Professional Psychologists (BDP), and the American Psychological Association (APA). It involved no invasive or otherwise ethically problematic techniques and no deception (and therefore, according to National jurisdiction, did not require a separate vote by a local Institutional Review Board; see the regulations on freedom of research in the German Constitution (§ 5 (3)), and the German University Law (§ 22)).

### Stimuli and procedure

Parents and their children visited the laboratory of the university for one experimental session. Children completed the optical illusion task, before both parents and children participated in a picture description task and an eye-tracking task. Children started with the tasks, while their parent was turned away from the scene, reading newspapers, and listening to music via shielded headphones. This was to prevent parents from seeing and hearing their children’s performance. Like their children, parents started with the picture description task, followed by the eye-tracking task. Note that children described pictures before parents described the pictures to them, in order to avoid direct situational influences (e.g., priming or imitation) from parental to descriptions on the descriptions of their children, but to draw conclusions about the result of cultural transmission processes more generally. The tasks analyzed in the present study were part of a more extensive data assessment.

#### Picture description task

Participants saw twenty real pictures that displayed objects (animals and means of transport), in front of a simple background (e.g., natural scenes, roads and buildings), see Fig 1 (pictures taken by the first author). Pictures taken by the authors were supplemented by pictures from a public domain database (pixabay.com). The pictures were presented on a 23-inch display, at a distance of about 70 centimeters, for 15 s each. Pictures were presented in a randomized order and separated by a blank screen. The psychophysics toolbox (Version 3.0.12) for MATLAB (Version R2008b) was used to present the pictures and to simultaneously record a 15 second audio file for later coding.

In the children’s version of the task, children were instructed to tell the experimenter, what they see on the pictures (exact wording: “…just tell me what you see on the pictures”). While children described the pictures, the experimenter motivated the children to describe the pictures with interested “mhm” sounds and nodding gestures, alternating the gaze between picture and the children.

In the adults’ version, parents were asked to describe the pictures to their children, while their children sat beside them and listened silently. Beforehand children were told that their parents (being turned away wearing headphones) did not know that the children had seen the pictures already and that they should keep this a secret, while listening to their parents’ descriptions silently. This was to motivate the children to listen to their parents and to prevent them from interrupting their parent while describing the pictures.

#### Eye-tracking task

Both children and parents saw eighty pictures. First, 40 real, semantic pictures which displayed objects in front of a simple background [3]. These pictures were structurally equivalent to the stimuli from the picture description task and were taken from the same sources. Thereafter, 40 abstract, non-semantic pictures with abstract objects in front of abstract backgrounds were shown, see Fig 1A and 1B. We used artificial objects commonly used in experimental psychology [20] (greebles, fribbles, geons and multipart geons, taken from an online database: http://wiki.cnbc.cmu.edu/Novel_Objects, available under GNU Free Documentation License 1.3). Abstract backgrounds were either fractal pictures [21] (20 pictures, created with quadrium 2.0, quadrium.en.softonic.com) or details of an abstract drawing (20 pictures), produced by the authors and students. The pictures of both sets were presented in a randomized order.

The instruction for the children, and later on the parents, was to “…look at the pictures attentively…”. While the parent watched the pictures, a second experimenter watched picture books with the children.

Trials started with a fixation dot (shown for 1 s), followed by the stimulus (5 s). The pictures were presented on a 23-inch display, at a distance of about 70 centimeters, covering an average visual angle of about 40.2 × 23.0°. Participant’s eye movements were recorded by a remote eye-tracking unit (redm 250; SensoMotoric Instruments GmbH, Teltow, Germany), binocularly, at a sampling rate of 60 Hz or higher. To calibrate the eye-tracker, participants made saccades to a grid of nine fixation dots on the screen and four dots were used to validate the calibration results.

#### Optical illusion task

We used four optical illusions (Müller-Lyer illusion, two versions of the Ebbinghaus illusion, Sander illusion), where the adjustable element was colored in red and the contextual elements were colored in gray, see Fig 1C. Each illusion was shown twice, i.e., the red element could be adjusted once on each side, resulting in eight trials, shown in a randomized order. In each trial, children could adjust the red element so that the two red elements are of equal size. To ensure that children understood the size should be adjusted in absolute size, but not in relative size to the different gray context elements, children were asked to pay attention to the red elements only. Prior to the actual task, children were instructed carefully and could practice the adjustment of a red element in one training trial.

Furthermore, before the optical illusion task, children adjusted the size of the pair of red elements of all eight trials (as well as the training trial) without the presence of the gray context element. These data were used to compute the degree to which children were deceived by the context.

Stimuli were presented on a 23-inch Monitor. The red element that could be adjusted was indicated by a black arrow and could be adjusted between -20 and +20 percent, compared to the size of the reference element, in steps of 1 percent, via two keys of a keyboard.

### Data analysis

#### Picture description analysis

The 15 s audio recordings for each of the twenty trials of the parents and the children were imported into MaxQDA (Version 12), for the coding of the audio track. Each occurrence of the following categories was coded: (a) Focal Object: References to the focal object (e.g. camel, car) and its features (e.g., is large, looks happy), (b) Background: References to the background (e.g., desert, road) and its features (e.g., is rocky, has green leaves), and (c) Relations: Any relations between elements within the picture (e.g., is driving on, is looking at). The mean number of relations mentioned when describing a picture were used as the score for the analyses. Inter-rater agreements for the frequencies per code and picture were assessed for 25% of the data (Cohen’s kappa: κ _focal object_ = .91, κ _object features_ = .92, κ _background_ = .94, κ _background features_ = .92, κ _relations_ = .95).

In order to quantify participants’ descriptions of the object compared to the background, we computed an object score: For each trial, all references to the object and its features were summed and divided this number by the number of all references to the object and the background, including all features. Thus, a score of 1 would indicate that a participant does only talk about the object, while a score of 0 would indicate that a participant does only refer to the background. These scores were averaged over all trials for each participant. We used the object score and the mean number of relations (averaged over trials) mentioned by the participants as indicators for children’s and parent’s context-sensitivity.

#### Eye-tracking analysis

The ExperimentCenter (Version 3.5.169, SensoMotoric Instruments GmbH, Teltow, Germany) was used to define regions of interest (ROIs) around the focal object of each picture and individual fixations were identified by a Low Speed Event Detection filter implemented in BeGaze (Version 3.5.101). Fixations were then exported for further analyses in MATLAB (Version 2013a). To quantify participants’ visual attention to the object, relative to the visual attention for the context, we calculated an object score: We summed the duration of all fixations made into the ROI of the object and divided this by the duration of all fixations on the picture within the 5 s of stimulus presentation. Thus, a score of 1 would indicate that the participant does only look at the object, while a score of 0 would indicate that a participant does only look at the background. We applied the same relative object score to the gaze data of 100 ms bins to track the temporal course of the object focus throughout the stimulus presentation time.

#### Optical illusion analysis

Children’s illusion score was computed by subtracting the percent of deviation in the optical illusion tasks from the percent of deviation in the trials without contextual information. The mean overall eight trials was used as the context-sensitivity score for this task.

#### Statistical analysis

T-tests were used to compare the picture description scores and gaze behavior of parents and children. Furthermore, we used Pearson’s correlations within the samples of children and parents and to analyze the relation between the context-sensitivity scores from parents and children and between the measures of children and parents picture description scores (objects score and relations) to test the relation between the verbal contexts-sensitivity scores. Because parental picture descriptions should be positively associated with children’s picture description scores, one-sided *p*-values are calculated for these correlations. We tested multiple directed hypotheses about the correlations between context-sensitivity measures in the adult sample (two verbal and two eye-tracking measures), we conducted one-sided tests, and applied a false discovery rate (FDR [22]) adjusment to the p-values of the six directional hypotheses. Furthermore, we checked for the influence of multiple comparisons on the relation between parent-child correlations. Note that we did not have any strong hypotheses about the correlations between context-sensitivity scores of children and those *p*-values are uncorrected and should be understood descriptively. All data reported in the manuscript are available in the Supporting Information (S1 File).

## Results

### Context-sensitivity scores of parents and children

In the picture description task, children made an average of 3.48 (*SD* = 1.54) references to the object or the background, significantly less than their parents, who made 6.14 (*SD* = 1.36) references, *t* = 6.86, *p* < .001. However, the object score, i.e., the relative emphasis on the object in relation to the background, did not differ between parents and children, see Table 1. In most trials, children (*M* = 92.2%, *SD* = 10.2%) and parents (*M* = 93.2%, *SD* = 5.2%) started with the focal object or its features.

**Table 1:** Context-sensitivity scores of parent and child.

Task and measure	Parent	Child	*t*	*p*
Picture description task				
Object score	.53(.10)	.51(.28)	.46	*n*.*s*.
Relations	.30(.09)	.08(.10)	10.00	p < .001
Eye-Tracking task				
Real Pictures	.56(.09)	.72(.06)	-6.86	p < .001
Abstract Pictures	.41(.14)	.50(.15)	-2.17	p < .05

Note: The table presents means with standard deviations in parentheses. T-values are presented along with two-sided p-values. Higher context-sensitivity is indicated by higher scores for “relations”, but by lower scores for all other measures.

**Fig 2:**
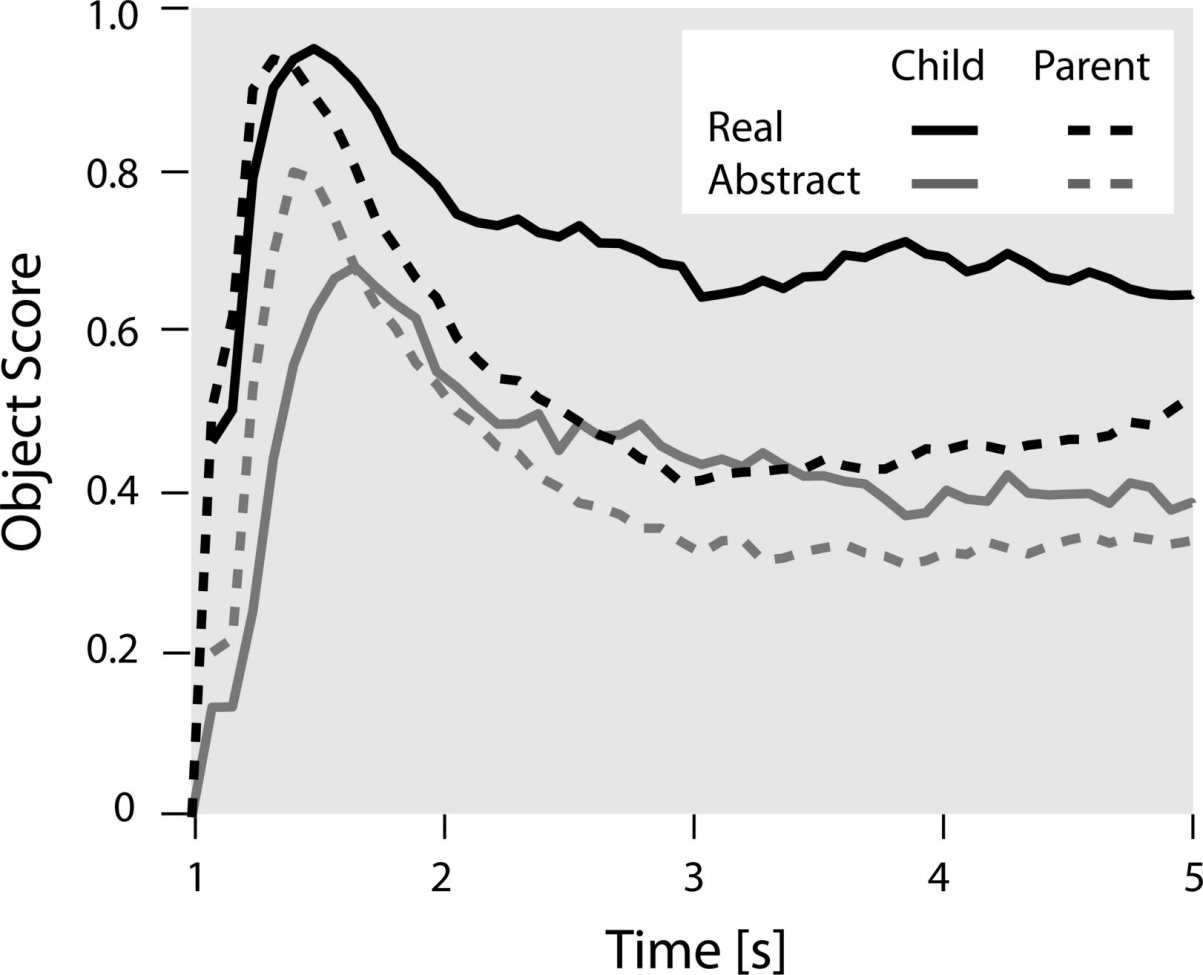
Temporal course of participants’ visual attention. Context-sensitivity in the eye-tracking task throughout the 5 s of stimulus presentation. The object score indicates the relative number of fixations made to the focal object, relative to all fixations on the picture (object and background), in 100 ms time bins. Separate lines indicate the object focus for parents and children on real and abstract scenes (see Fig 1).

For the eye-tracking task, the relative amount of time spent to explore the focal object in real and abstract pictures, i.e., the object scores, are given in Table 1. Parents spent about half of the time exploring the object in both normal and abstract scenes. Children spent more time looking at objects in real as well as in abstract pictures. Fig 2 displays the time course of the visual exploration for parents and children. Similar to the picture description task (see above), children and parents initially directed their attention to the focal object before exploring the background.

In the optical illusion task, children were deceived on average by 11.9% (*SD* = 4.4), *M* = 17.3% (*SD* = 8.4) in the Müller-Lyer illusion, *M* = 7.0% (*SD* = 6.9) across the two versions of the Ebbinghaus illusion, and *M* = 16.3% (*SD* = 7.2) in the Sander illusion.

### Correlations among the context-sensitivity scores

For parents, picture description scores, namely the object score and the number of relations, were correlated negatively, i.e., the more the description was focused on the background, the more relations were mentioned (See Table 2). Picture description scores were further related to parental gaze behavior in the eye-tracking paradigm. In particular, the more parents focused on the object when describing the pictures, the more time they spent fixating the object in both normal and abstract pictures. Consistently, the more relations parents mentioned, the more time they spent fixating the background of the abstract pictures in the eye-tracking task, i.e., the number of relations and the abstract picture score correlated negatively. Finally, context-sensitivity measured in the eye-tracking task were consistent across normal and abstract pictures, as indicated by a significant correlation between both scores.

**Table 2:** Correlations among context-sensitivity scores of the parents.

Task and measure	1.	2.	3.
Picture description task			
1. Object score			
2. Relations	-.54**		
Eye-Tracking task			
3. Real Pictures	.40*	-.25^(^*^)^	
4. Abstract Pictures	.55**	-.46**	.60**

Note. The table displays Pearson’s correlation coefficients (r).

^(^*^)^ p = .10

* p < .05

** p < .01

*** p < .001, one-sided, FDR adjusted.

For children, the picture description scores, i.e., the object score and the number of relations, were neither correlated to each other, nor to the eye-tracking scores (see Table 3). Likewise, no correlation was found between the context-sensitivity scores assessed for normal and abstract pictures. The score from the optical illusion task was negatively correlated with the object score of the picture description task and there was a tendency for a positive correlation between the optical illusion score and the number of relations mentioned by the child. However, these *p*-values would not survive multiple comparisons and should thus be understood descriptively.

**Table 3:** Correlations among context-sensitivity scores of the children.

Task and measure	1.	2.	3.	4.
Picture description task				
1. Object score				
2. Relations	-.16			
Eye-Tracking task				
3. Real Pictures	.08	.21		
4. Abstract Pictures	-.22	-.03	.00	
Optical illusion task				
5. Illusion score	-.37*	.25^(^*^)^	.20	-.06

Note. The table displays Pearson’s correlation coefficients (r).

^(^*^)^ p < .10

* p < .05, one-sided, uncorrected.

### The relation between picture description accounts of parents and children

**Fig 3:**
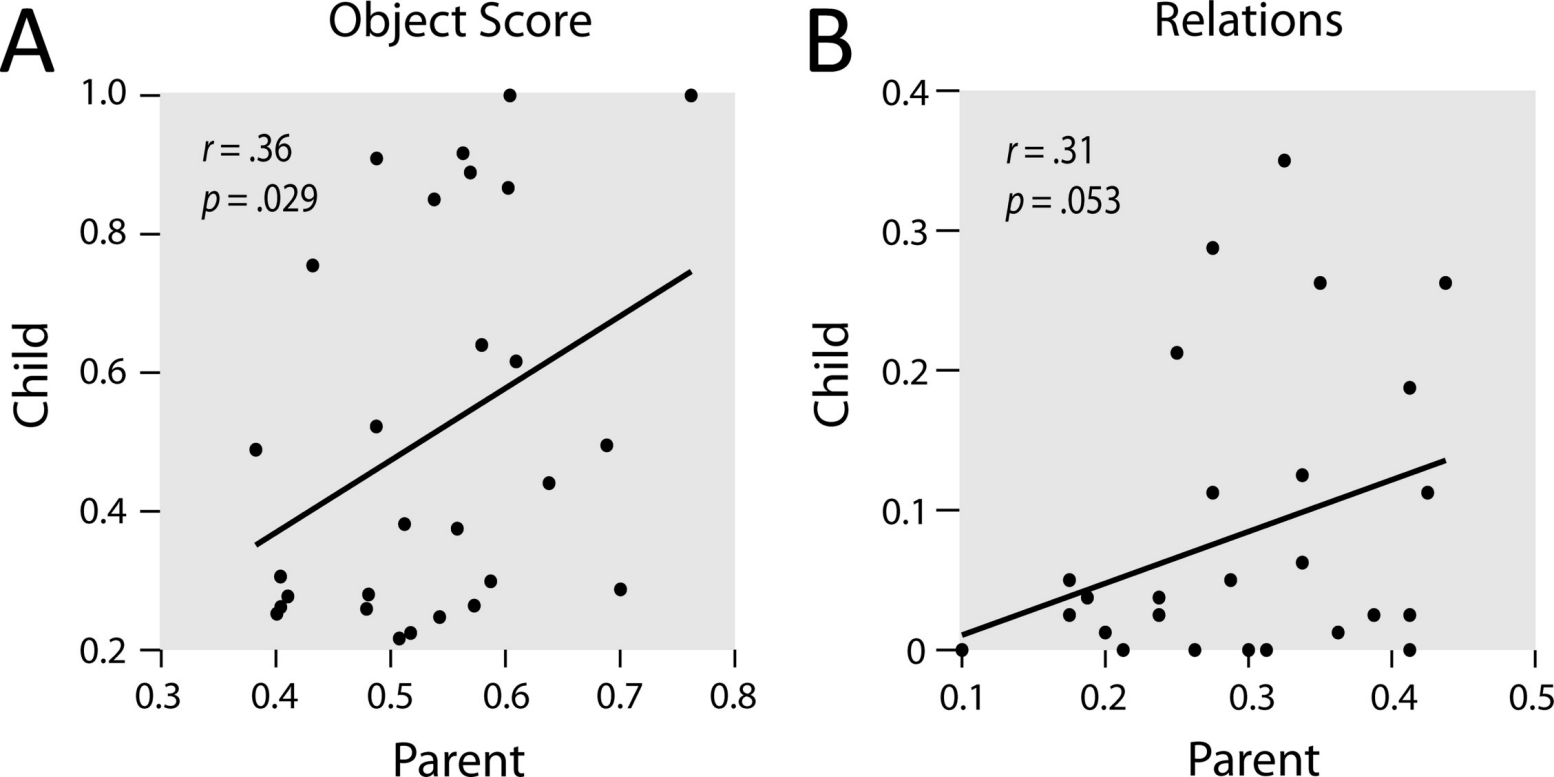
Relation between parents’ and children’s context-sensitivity assessed in the picture description task for the object score (A) and relations (B) The results of the correlational analyses are displayed in the graph, with one-sided *p*-values. FDR adjusted *p*-values are both *p* = .053.

In the picture description task, parents’ object scores were positively correlated with children’s object scores, *r* = .36, *p* = .029, one-sided. Furthermore, there was a marginal trend for a positive correlation between the mean number of relations mentioned by parents’ and children, *r* = .31, *p* = .053, one-sided (see Fig 3). When corrected for multiple comparison, both relations were at the level of a marginal trend, both *p* = .053. Critically, these relations were not due to similarities in talkativeness of parents and children, that is, their total number of codes, including references to the object, the background and all features were not correlated, *r* = .02, *p* = .940. Furthermore, no correlations were found between gaze parameters of parents and children (all |*r*| < .16, all *p* > .416).

## Discussion

Importantly, the way in which children described visual scenes was significantly associated with the way in which parents described these scenes to them, focusing on either focal objects and their features or emphasizing contextual features and relations between elements of a scene. This supports our main hypothesis that children’s context-sensitivity is socialized via a verbal route in the parent-child interaction.

For adults, we found significant associations (1) between the picture description scores and visual attention measures, (2) between both scores of the picture description task, i.e., the object score and number of relations, and (3) between the visual attention pattern in real and abstract scenes. Children’s context-sensitivity in the picture description task was not associated with their gaze behavior in real or abstract scenes. However, their picture description accounts were related to their degree of deception in the optical illusion task. Thus, children’s perception was consistent across the two behavioral tasks, but their context-sensitivity was not related to their visual attention processes. Thereby, the present study provides empirical evidence that behavioral measures of context-sensitivity are related to visual attentional processes in adults, but not yet in children.

These are important additions to the existing literature and promote the understanding of the ontogenetic development of context-sensitivity and its different facets. These findings are in line with recent evidence that visual cortical networks (measured in the electroencephalogram) restructure and align with verbal measures of context-sensitivity after the preschool years [19]. While there is evidence for cross-cultural differences already at an earlier age [10–15], these former studies did not scrutinize the relations between different measures. However, consistent relations between different indicators of context-sensitivity are a critical measure for the emergence of culture-specific perception styles. For instance, a recent study on 5-year-olds from three cultures found a number of cross-cultural differences but no consistent patterns or relations between different indicators of context-sensitivity [9]. Thus, it is difficult to generalize perception styles from single measures. It would be valuable to extend the present findings in further cultural contexts. Although we would expect very similar relations between parental socialization and child’s scene description like in the present study, these findings would substantiate the idea that parental guidance of attention is a universal principle that underlies culture-specific developmental trajectories [10, 16]. Furthermore, given that the findings of the present study are correlational, it would be valuable to substantiate the socialization of context-sensitivity with experimental manipulations of the teaching style or longitudinal approaches.

Taken together, the present findings suggest that context-sensitivity is socialized via a verbal route in the preschool years and socialization experiences influence verbal processes first, before visual attention aligns with language, to form a unitary construct that organizes scene descriptions, perception, and visual attention in individual, and putatively culture-specific, ways. That the scene descriptions of children were similar to those of the parents in two important aspects (relative focus on focal objects, the number of relations uttered) provides convergent evidence for the idea that children acquire cultural meaning systems through social exchange in the form of narratives [23]. The late emergence perspective on context sensitivity is compatible with the more general theoretical frameworks, which assume that context-sensitivity is tightly knit to personal development, such as culture-specific self-construals [24].

## Supporting information

S1 File Data file. (SAV)
